# Communication molecules (ncRNAs) mediate tumor-associated macrophage polarization and tumor progression

**DOI:** 10.3389/fcell.2024.1289538

**Published:** 2024-03-08

**Authors:** Min Yao, Xuhua Mao, Zherui Zhang, Feilun Cui, Shihe Shao, Boneng Mao

**Affiliations:** ^1^ The Affiliated Yixing Hospital of Jiangsu University, WuXi, China; ^2^ School of Medicine, Jiangsu University, Zhenjiang, China; ^3^ The Affiliated Taizhou Second People`s Hospital of Yangzhou University, Taizhou, Jiangsu, China

**Keywords:** ncRNAs, TAMs, polarization, tumor progression, signaling pathways

## Abstract

Non-coding RNAs play important roles in tumor cells and macrophages and participate in their communication as messengers. Non-coding RNAs have an impact in tumor cell proliferation, migration, and apoptosis, and they also regulate the differentiation and regulation of immune cells. In macrophages, they stimulate the polarization of macrophages into M1 or M2 by regulating proteins related to signaling pathways; in tumor cells, non-coding RNAs can enter macrophages through exosomes and affect the latter polarization. The polarization of macrophages further regulates the biological functions of cancer cells. The direction of macrophage polarization determines tumor progression, angiogenesis and drug resistance. This often creates a feedback loop. Non-coding RNAs act as bridges between tumor cells and macrophages to regulate the balance of the tumor microenvironment. We reviewed the signaling pathways related to macrophage polarization and the regulatory mechanisms of non-coding RNA in tumor-associated macrophages M1 and M2, and discussed the potential applications and prospects of exosome engineering.

## 1 Introduction

Non-coding RNAs (ncRNAs) are RNA molecules that do not code for proteins. They play a crucial role in gene regulation and have been related to a variety of cellular processes, including development, cell differentiation, and cell death (Toden et al., 2021; Yan and Bu, 2021; Toden and Goel, 2022). Presently, predominant investigations revolve around micro RNAs (miRNAs), long non-coding RNAs (lncRNAs), and circular RNAs (circRNAs). miRNAs are short RNA molecules, typically consisting of approximately 21–25 nucleotides, that regulate gene expression by binding to complementary sequences in the 3′untranslated regions (UTRs) of target mRNAs (Vishnoi and Rani, 2023). This binding results in mRNA degradation or translational repression, thereby controlling the abundance of specific proteins. miRNAs play important roles in a variety of cellular processes, including development, differentiation, apoptosis, and stress responses (Liu et al., 2023). Moreover, their implication extends to pathological conditions such as cancer and neurodegenerative disorders.

Long non-coding RNAs (lncRNAs), exceeding 200 nucleotides in length, encompass a broad array of biological roles (Bridges et al., 2021). These encompass epigenetic orchestration, chromatin manipulation, transcriptional governance, and protein complex scaffolding (Herman et al., 2022). Some lncRNAs act as guides for chromatin-modifying complexes to target specific genomic loci. lncRNAs are involved in various cellular processes such as X chromosome inactivation, imprinting, and regulation of gene expression (Chanda and Mukhopadhyay, 2020). CircRNAs are covalently closed single-stranded RNA molecules that form circular structures due to a unique splicing event called back-splicing (Li, 2023). The function of circRNAs is still being explored, but they are thought to act as regulators of gene expression through the miRNA sponge mechanism (Liu et al., 2022). CircRNAs’ participation spans diverse cellular processes, encompassing domains such as cerebral function, immune responsiveness, and oncogenic pathways (Mehta et al., 2020; Li B. et al., 2021). The cyclic nature of circRNAs imparts enhanced stability when contrasted with linear RNA counterparts (Hoque et al., 2023).

Macrophages are immune cells present in all tissues and play a crucial role in both immune responses that are innate and adaptive. The conventional perspective asserts that macrophages primarily derive from monocytes (mo-macs), while emerging evidence also signifies their embryonic origin (resident tissue macrophages, RTMs) (Jian et al., 2023). mo-macs originate from hematopoietic stem cells (HSCs) and exhibit distinct functional and phenotypic attributes in comparison to RTMs. The differentiation trajectory of mo-macs involves sequential transitions from lymphoid-myeloid progenitors (LMP) to granulocyte-macrophage progenitors (GMP), culminating in monocytes, which subsequently migrate to diverse tissues to mature into macrophages (Terry and Miller, 2014; Shi et al., 2021). RTMs originate from the yolk sac and fetal liver embryonic precursors that infiltrate these tissues during embryogenesis, yielding self-sustaining, locally proliferating tissue-resident macrophage populations that endure into adulthood (Ginhoux and Guilliams, 2016; Vaughan-Jackson et al., 2022; Bied et al., 2023). However, in other tissues such as the heart, pancreas, or gut, some RTM may also originate from circulating monocytes, possibly because embryonic RTM cannot keep up with the tissue’s demand for phagocytes, but this requires prolonged cell-tissue Microenvironmental interactions (Chakarov et al., 2019; Blériot et al., 2020). This review mainly emphasizes a comprehensive synthesis of the study of ncRNAs in macrophages in the context of tumor biology (Table 1). They are involved in defense against pathogens, tissue repair and maintenance of tissue homeostasis. Macrophages are also involved in the initiation and progression of cancer (Chen et al., 2019; Li C. et al., 2021; Lopez-Yrigoyen et al., 2021). In the context of cancer, macrophages can have both pro-tumor and anti-tumor properties. By promoting angiogenesis, inhibiting anti-tumor immune responses, and providing a supportive microenvironment for tumor cells, macrophages can promote tumor growth and progression (Mantovani et al., 2002; Chen et al., 2017; Zhang et al., 2019). On the other hand, macrophages can also play an anti-tumor role by promoting immune-mediated tumor cell death and inhibiting tumor angiogenesis (Xu et al., 2018; Cheng et al., 2022; Zhao et al., 2022). The function of macrophages in cancer is determined by their activation status, which is classified as pro-inflammatory (M1) and anti-inflammatory (M2). M1 macrophages are abundant in inflamed tissues and their gene expression profiles reveal high levels of pro-inflammatory cytokines such as TNF-α, MCP1, IL-6 and iNOS. MCP1 is a potent monocyte chemoattractant that interacts with membrane CC chemokine receptor 2 (CCR2) on monocytes to trigger monocyte chemotaxis and migration (Panee, 2012; Zhao et al., 2022). iNOS produces higher concentrations of nitric oxide (NO), which can lead to tissue damage leading to an inflammatory response since it is a potent vasodilator (Duan et al., 2023). In contrast, M2 macrophages are anti-inflammatory, activate STAT6 via the IL-4 receptor (IL-4Rα), and polarize via the Th2 cytokines IL-4 and IL13. In addition to IL-4, IL13, other cytokines such as IL10, IL-33, IL-21 can also control M2 polarization (Wang et al., 2014; Watanabe et al., 2017; Holgado et al., 2023; Mei et al., 2023). Due to their pro-angiogenic and pro-fibrotic characteristics, M2 macrophages frequently promote tumor growth. They also aid in tissue repair and wound healing. As a result, anti-inflammatory M2 macrophages are related to a pro-tumor phenotype while pro-inflammatory M1 macrophages are related to an anti-tumor phenotype (Pan et al., 2020; Razi et al., 2023). But classifying TAMs into distinct M1 or M2 categories is an oversimplification because the tumor microenvironment is highly dynamic and complex. TAMs can be influenced by a variety of factors, including cancer type, stage of tumor development, and the presence of other immune cells. As tumors develop, their function changes over time.

**TABLE 1:** Effects of ncRNAs on Macrophage Polarization in Tumors.

Type of cancer	ncRNA	Polarization direction	Target/pathways	Function	Ref.
4T1 breast cancer	miR-33	M1	—	miR-33 transform macrophage M2 into M1	Moradi-Chaleshtori et al. (2021)
Hepatocellular carcinoma	miR-144/miR-451a	M1	HGF/MIF	miR-144/451a cluster contributes to HCC progression via paracrine HGF/MIF-mediated TAM remodeling	Zhao et al. (2021)
Lung cancer	miRNA-let-7b-5p	M1	GNG5	miR-let-7b-5p regulates GNG5 to inhibit tumor cell proliferation and promote tumor cell apoptosis	Peng et al. (2023)
Glioma	miR-302a	M1	METTL3/SOCS2	miR-302a/METTL3/SOCS2 axis promotes the polarization of M1 macrophages and inhibits the development of glioma xenografts	Zhong et al. (2021)
Non-small cell lung cancer	miR-146a	M1	TRAF-6, IRAK-1	microRNA-146a promotes non-small cell lung cancer cell invasion and proliferation by inhibiting M1 macrophage polarization	Ren et al. (2021)
Hepatocellular carcinoma	miR-142-3p	M1	SLC3A2	miR-142-3p promote the progression of liver cancer by inducing iron death of M1-type macrophages	Hu et al. (2022)
Breast cancer	miR-138-5p	M1	KDM6B、H3K27me3	miR-138-5p regulates tumor-associated macrophage M2 polarization and promotes breast cancer progression	Xun et al. (2021)
Hepatocellular carcinoma	lncRNA GAS5	M1	PTEN	GAS5 overexpression promoted M1-like polarization of TAMs, thereby inhibiting cell proliferation and invasion by SMCC-7721 cells	Wang et al. (2020)
Hepatocellular carcinoma	lncRNA FTX	M1	—	Upregulation of lncRNA FTX suppressed NAFLD conversion to HCC though promoting M1 polarization of KCs	Wu et al. (2020)
Colorectal cancer	circPLCE1	M2	miR-485-5p/ACTG1	circPCLE1 expedites EMT, glycolysis in CRC and TAM M2 polarization via modulating the miR-485-5p/ACTG1 axis	Yi et al. (2022)
Triple-negative breast cancer	miR-34a	M2	MCT-1/miR-34a/IL-6/IL-6R	MCT-1/miR-34a/IL-6/IL-6R signaling axis promotes EMT progression, cancer stemness and M2 macrophage polarization in triple-negative breast cancer	Weng et al. (2019)
Breast cancer	miR-200c	M2	PAI-2	miR-200c/PAI-2 increased IL-10 expression and secretion in TNBC cells, ultimately promoting M2 macrophage polarization	Meng et al. (2020)
Breast cancer	miR-720	M2	GATA3	miR-720 affects breast cancer progression by regulating GATA3 and participating in M2 macrophage polarization	Zhong and Yi (2016)
Colorectal cancer	miR-934	M2	PI3K/AKT CXCL13/CXCR5/NFκB/p65	miR-934 induces macrophage M2 polarization to promote liver metastasis of colorectal cancer	Zhao et al. (2020)
Colorectal cancer	miR-222	M2	PTEN/AKT	miR-222 activated the Akt signaling pathway and polarized M2 macrophages, promoting breast cancer cell progression in a feedback loop	Chen et al. (2021a)
Bladder cancer	miR-21	M2	PTEN/AKT/STAT3	miR-21 activates the PI3K/AKT pathway in macrophages to promote bladder cancer progression	Lin et al. (2020)
Ovarian cancer	miR-217	M2	JAK3/STAT3、IL-6	miR-217 inhibits tumor-induced M2 macrophage polarization by targeting IL-6 and modulating JAK3/STAT3 signaling	Jiang et al. (2019)
Laryngeal squamous cell carcinoma	lncRNA HOTAIR	M2	PTEN, PI3K/AKT	lncRNA HOTAIR induce macrophages to M2 polarization via PI3K/p-AKT/AKT pathway and promote EMT and metastasis in laryngeal squamous cell carcinoma	Wang et al. (2022)
Thyroid cancer	lncRNA MALAT1	M2	FGF2	LncRNA MALAT1 mediates FGF2 protein secretion by TAMs and promotes FTC133 cell proliferation, migration, and invasion, and induces angiogenesis	Huang et al. (2017)
Hepatocellular carcinoma	lncRNA MALAT1	M2	miR-140	Knockdown of MALAT1 in HCC cells impaired the angiogenesis of HUVECs, and facilitated the polarization of macrophage toward the M1 subset.	Hou et al. (2020)
Gastric cancer	lncRNA H19	M2	miR-519d-3p/LDHA	H19 promotes aerobic glycolysis, proliferation, and immune escape of gastric cancer cells through the microRNA-519d-3p/lactate dehydrogenase A axis	Sun et al. (2021)
Colorectal cancer	lncRNA RPPH1	M2	TUBB3	LncRNA RPPH1 promotes colorectal cancer metastasis by interacting with TUBB3 and by promoting exosomes-mediated macrophage M2 polarization	Liang et al. (2019)
Colorectal cancer	ncRNA LCC-HOXB8-1:2	M2	miR-6825-5p/CXCR3/CXCL10	lncRNA lc-hoxb8-1:2 promotes TAM infiltration and M2 polarization through the miR-6825-5p/CXCR3/CXCL10 axis, thereby promoting the progression of neuroendocrine differentiation CRC.	Li et al. (2022)
Lung adenocarcinoma	miR-19b-3p	M2	LINC00273	miR-19b-3p facilitates M2 macrophage polarization and exosomal LINC00273 secretion to promote lung adenocarcinoma metastasis via Hippo pathway	Chen et al. (2021b)
Hepatocellular carcinoma	lncRNA TUC339	M2	—	Exosomal TUC339 from Hepatocellular carcinoma (HCC) regulates M2 macrophage activation and polarization	Li et al. (2018)
Hepatocellular carcinoma	LINC00662	M2	Wnt/-catenin	LINC00662 promotes M2 macrophage polarization and HCC progression by activating Wnt/-catenin signaling	Tian et al. (2020)
Hepatocellular carcinoma	SNHG20	M2	STAT6	Overexpression of SNHG20 stimulates the activation of STAT6, induces M2 polarization of hepatic KCs, and promotes NAFLD progression to HCC.	Wang et al. (2019)
Esophageal cancer	circRNA TCFL5	M2	FMNL2/miR-543	CircRNA TCFL5 promotes esophageal cancer cell proliferation, invasion, and migration by regulating the FMNL2/miR-543 axis, mediates macrophage M2 polarization, and promotes tumor growth *in vivo*	Lin et al. (2022)
Ovarian cancer	circATP2B4	M2	miR-532-3p/SREBF1/PI3K/AKT	CircATP2B4 promotes M2-type polarization of macrophages through miR-532-3p/SREBF1/PI3K/AKT signaling pathway, leading to immunosuppression and ovarian cancer metastasis	Wang et al. (2023)
Breast cancer	circ 0001142	M2	miR-361-3p/PIK3CB	Exosomal circ 0001142 promoted the M2 polarization of macrophages and increased the proliferation and metastasis of breast cancer cells via the miR-361-3p/PIK3CB axis	Lu et al. (2022)
Breast cancer	CircWWC3	M2	IL-4/PI3K/STAT6	CircWWC3 augments breast cancer progression through promoting M2 macrophage polarization and tumor immune escape via regulating the expression and secretion of IL-4	Zheng et al. (2022)

Macrophage metabolism is closely related to activation phenotype. Polarization between M1 and M2 macrophages requires different energy sources and metabolic pathways. During IFN-γ/LPS-activated macrophage M1 polarization, glycolytic bursts and the pentose phosphate pathway (PPP) are able to rapidly produce the necessary energy, but during IL-4-induced macrophage M2 polarization, metabolism is mainly dependent on more sustainable energy sources (Jones et al., 2021). Such as mitochondrial respiration (oxidative phosphorylation [OXPHOS]) and fatty acid oxidation (FAO). In addition, the adjustment of mitochondrial metabolism, including changes in oxidative metabolism, mitochondrial reactive oxygen species (mtROS), mitochondrial ultrastructure and membrane potential, contributes to different states of macrophage activation (Roca et al., 2022; Stifel et al., 2022). Therefore, interference with macrophage energy metabolism (glycolysis and mitochondrial activity) can alter the phenotype and function of macrophages, thereby affecting the host immune response (Dussold et al., 2024). Within the tumor microenvironment (TME), tumor progression is accompanied by increased hypoxic conditions, nutrient deficiencies, and the accumulation of several cancer-cell derived metabolites and metabolic byproducts. These metabolites influence the properties of TME, which is also involved in the regulation of cellular metabolism, and this dynamic communication persists during the development of cancer. Therefore, understanding the regulation and function of macrophages in cancer is crucial for developing effective anticancer therapeutic strategies.

**FIGURE 1:**
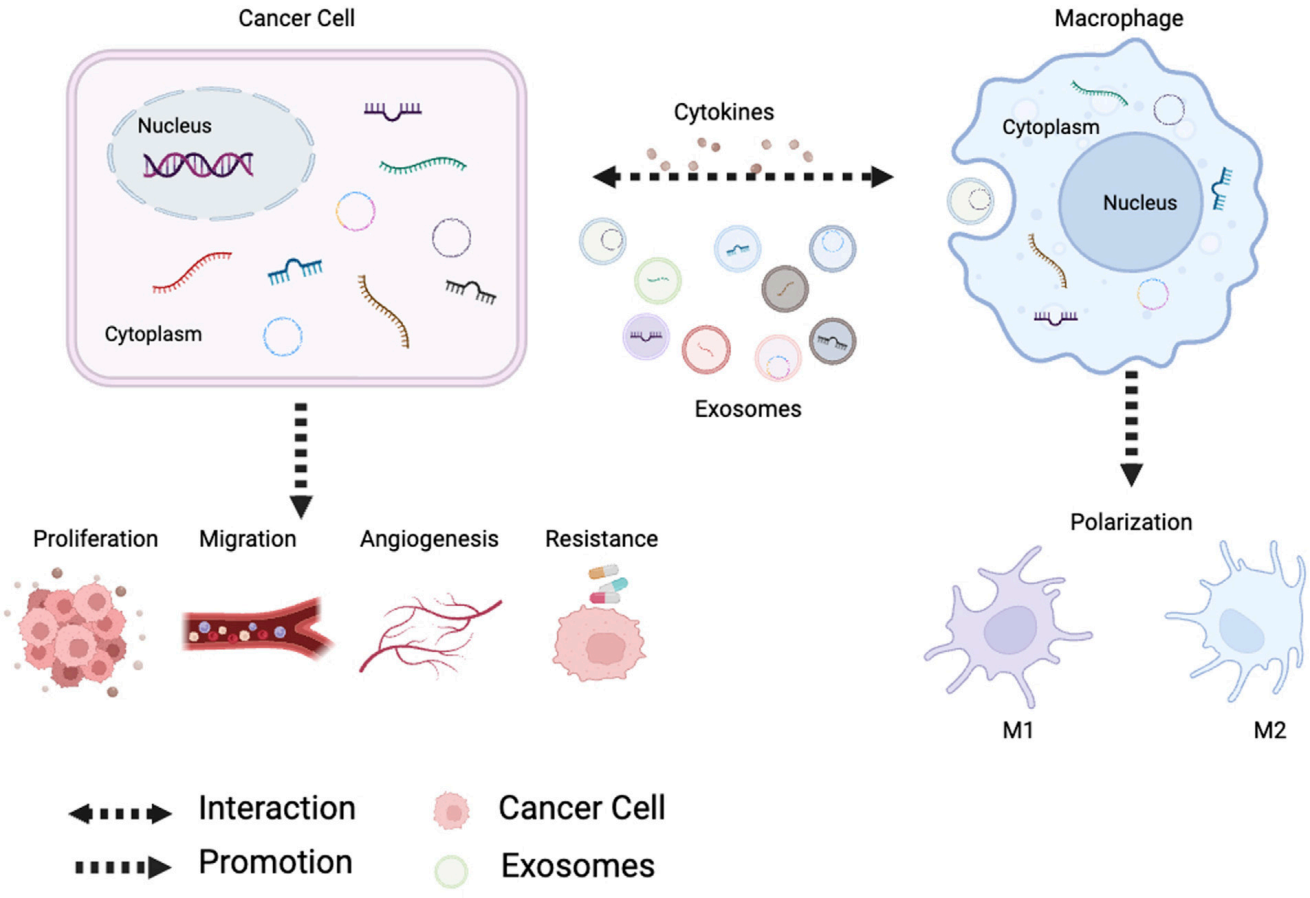
Tumor-derived noncoding RNAs regulate macrophage M1/M2 polarization through exosomes and cytokines. Polarized macrophages affect tumor proliferation, metastasis, angiogenesis and chemoresistance.

Many basic and preclinical experiments have shown that ncRNAs have become important players in the regulation of cancer gene expression. As communication molecules between tumor and immune cells, ncRNAs are actively involved in tumor-associated macrophage (TAMs) polarization, and polarized TAMs regulate the malignant phenotype of cancer cells, which often forms a feedback loop (Figure 1). The purpose of this review is to summarize the role of ncRNAs in TAMs and cancer.

## 2 Crosstalk of signaling pathways during macrophage polarization

In macrophage polarization, multiple signaling pathways and transcriptional and post-transcriptional regulatory networks are involved, including PI3K/AKT signaling pathway, Janus kinase/signal transducer and activator of transcription (JAK/STAT) pathway, nuclear factor-kappa B (NF -κB) pathway and peroxisome proliferator-activated receptor gamma (PPAR-γ) pathway. The most studied and thoroughly studied pathway is the PI3K/AKT signaling pathway, is activated in macrophages in response to various stimuli such as growth factors and cytokines. JAK/STAT signaling pathway appears to be particularly important during M1-type polarization. During the inflammatory response, Under the action of IL-12, CD4+T cells develop in the direction of Th1 through upregulation of the transcription factor T-Bet, and IL-12 promotes the production of INF-γ by promoting the activation of STAT4 and T-Bet (Yunna et al., 2020). INF-γ activates the JAK family of tyrosine kinases upon binding to the receptor, which in turn phosphorylate signal transducers and activators of transcription (STATs) protein (Zhang et al., 2017). Regarding phosphorylation, STAT proteins move into the nucleus where they bind to specific DNA sequences to control gene expression, causing macrophages to polarize toward the M1 form. NF-κB signaling pathway is also involved in the M1 polarization of macrophages. Mediated by LPS, Toll-like receptor 4 (TLR4) activates PI3K through BCAP adapter protein binding to PI3K and generates tumor necrosis factor-related factor receptor 6 (FRAF6) (Safaroghli-Azar et al., 2023). FRAF6 can not only promote the activation of NF-κB/AP-1 transcription factor to induce M1 polarization, but also directly recruit and activate Stat1, thereby inducing macrophages to polarize to M1 (Gorina et al., 2011). The activities of these pathways are interrelated because activation of the JAK/STAT pathway can result in phosphorylation of the NF-κB inhibitor IκBα, resulting in the release of NF-κB from the NF-κB/IκBα complex and activation of rapid translocation to the nucleus and further induces pro-inflammatory genes (Van Dyken and Locksley, 2013). This interplay between the JAK/STAT and NF-κB pathways provides a positive feedback loop that amplifies the inflammatory response in M1 macrophages.

CD4+T cells are induced to differentiate into Th2 by TCR stimulation under the action of IL-4. IL-4 not only enhances Th2 differentiation by blocking IL-12 and IFN-γ, but also activates STAT6 to induce the expression of transcription factor GATA3, which can promote Th2 cell differentiation and help maintain Th2 phenotype (Jablonski et al., 2015). During M2-type polarization, Th2 cells secrete IL-4 cytokines and bind IL-4R, activate JAK1 and JAK3, recruit the adapter protein IRS2 and simultaneously activate Stat6 and lead to its phosphorylation (Marques-Ramos and Cervantes, 2023; Zhang and Sioud, 2023). On the one hand, IRS2 binds to PI3K, and the second messenger of PI3K is phosphor phosphate molecule 4.5-dioxide (PIP2), which is phosphorized to produce phosphate molecules 3.4-triphosphates (PIP3) . PIP3 then phosphorylatese mTORC2 and activate AKT. Activated AKT phosphorylates and inactivates the tuberous sclerosis complex (TSC). Inactivation of TSCs leads to activation of the target of rapamycin complex 1 (mTORC1), which polarizes cells towards M2 (Bansal et al., 2015; Liu and Sabatini, 2020; Melick and Jewell, 2020). On the other hand, activated STAT6 can bind to macrophage-like factor 4 (KLF4) and peroxisome proliferator-activated receptor (PPAR)-γ to promote M2-type polarization (Guo et al., 2022).

**FIGURE 2:**
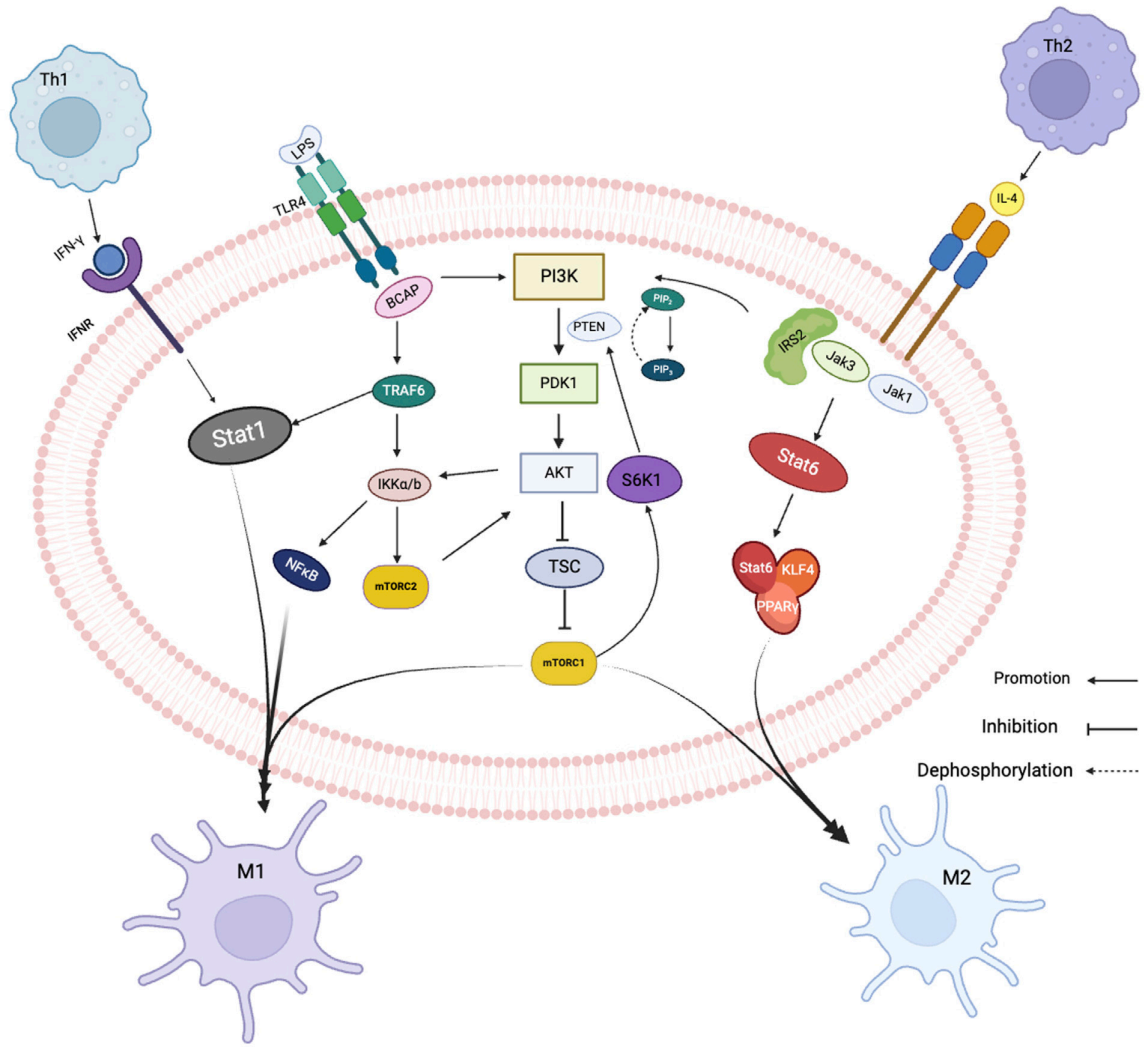
Related signaling pathways of macrophage polarization. Black arrows show signal direction, T-bars indicate inhibition of subsequent signaling, dotted arrows dephosphorylation.

In summary, the PI3K/AKT, JAK/STAT, and NF-κB signaling pathways are connected in series to form a complex network of crosstalk and regulation during macrophage polarization. The balance between these pathways is critical for the proper polarization of macrophages towards the M1 or M2 phenotype (Figure 2).

## 3 The role of ncRNA on the M1 polarization of tumor-associated macrophages

In triple-negative breast cancer, the tumor suppressor miR-34a suppresses the expression of IL-6R and attenuates the role of the MCT-1 gene in epithelial-mesenchymal transition (EMT). MCT-1 induces angiogenesis and tumor evasion from immune surveillance by stimulating interleukin 6 (IL-6) secretion, and downregulation of its expression suppresses M2 macrophage polarization and increases M1 macrophage polarization (Weng et al., 2019). The secretion of miR-33 exosomes from 4T1 breast cancer cells can transform macrophage M2 into M1 type, and the secretion of M1-specific cytokines TNF-α and IL-1β is increased, while the secretion of M2-specific cytokines IL-10 and TGF-β is decreased (Moradi-Chaleshtori et al., 2021). In clinical samples of hepatocellular carcinoma, the expression of miR-144 and miR-451a was correlated positively with M1 polarized macrophage marker HLA-DR. *In vitro* experiments showed that miR-144 and miR-451a promoted M1 polarization in the tumor microenvironment and inhibit HCC growth by targeting HGF and MIF (Zhao et al., 2021). miR-let-7b-5p from macrophage M1 is transported to lung cancer cells through exosomes, regulates GNG5 protein level to inhibit tumor cell proliferation and promote tumor cell apoptosis (Peng et al., 2023). *In vivo* and *in vitro*, 1C-containing Jumonji domains promote M1 macrophage polarization and inhibit the development of glioma xenografts via the miR-302a/METTL3/SOCS2 axis (Zhong et al., 2021). Non-small cell lung cancer cells (NSCLC) secrete exosomal miR-146a, which inhibits TRAF-6 and IRAK-1 expression in TAMs (Yan et al., 2022). TRAF6 is an essential component of the M1 polarization process. It not only activates NFκB signaling pathway by inducing IKKa/β phosphorylation, but also directly interacts with Stat1 to promote M1 polarization of macrophages (Ren et al., 2021). Decreased expression of TRAF-6 resulted in impaired antitumor activity of TAMs, thereby promoting invasion and proliferation of NSCLC cells. In HBV-positive HCC cells, exosomal miR-142-3p promote HCC progression in M1 macrophages by inducing ferroptosis (Hu et al., 2022). miR-138-5p is delivered by exosomes from breast cancer cells to tumor-associated macrophages to downregulate KDM6B expression (Xun et al., 2021). KDM6B controls gene expression in LPS-activated macrophages through H3K27me3, and its downregulation inhibits the recruitment of M1-associated gene promoters (De Santa et al., 2009).

A small number of lncNAs were found to be associated with M1 macrophages and have antitumor effects. GAS5 expression was higher in M1 macrophages but lower in M2 macrophages. GAS5 overexpression increases the PTEN expression in TAMs, inhibits M2 polarization and promotes M1 polarization by inhibiting the activity of AKT signaling pathway. The TAM supernatant treated with silencing PTEN can enhance the proliferation and invasion ability of SMCC-7721 cells while reducing the effect of GAS5-overexpressing TAM supernatant on SMCC-7721 cells. These results suggest that GAS5 overexpression inhibits the M2-like polarization of TAMs by enhancing PTEN expression, thereby inhibiting liver cancer progression (Wang et al., 2020).

**FIGURE 3:**
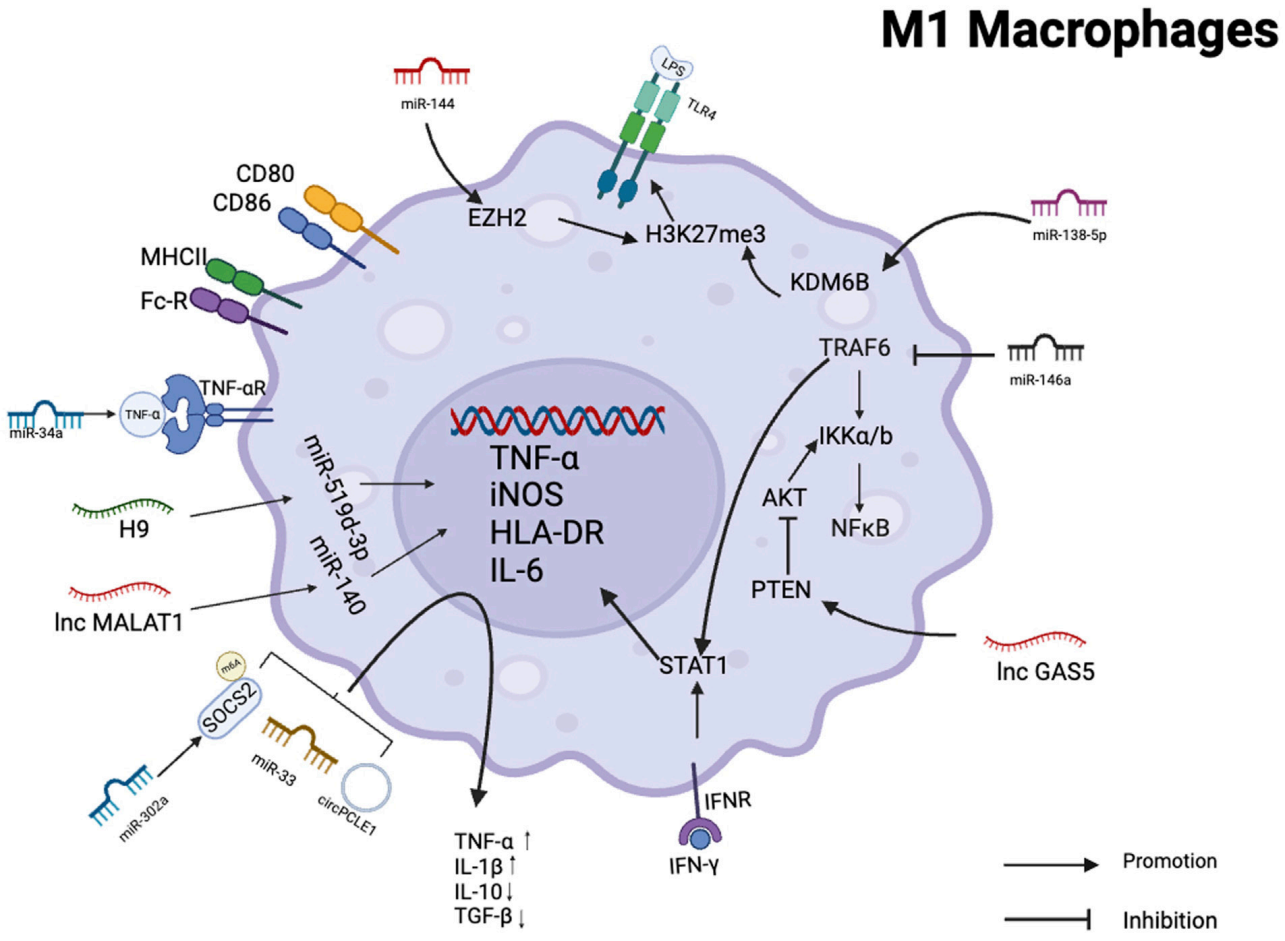
Schematic summary of the role of various non-coding RNAs in regulating M1 polarization in macrophages. This schematic illustrates the role of ncRNAs as effectors of macrophage M1 activation and polarization in several human cancer cells.

Reduced circPCLE1 expression inhibits M2 macrophage markers (IL-10, MRC1), glucose consumption, and cell proliferation in colorectal cancer, while increasing the proportion of M1 macrophage markers (TNF-, IL-6) and decreasing the CD206+ and CD168+ macrophages. This suggests that circPCLE1 restricts the biological functions of colorectal cancer and facilitates the polarization of TAMs towards M1 (Yi et al., 2022) (Figure 3).

## 4 Role of ncRNAs in M2 polarization of tumor-associated macrophages

NcRNAs are critical regulators of M2 macrophage polarization and function. Although the miR-200c/PAI-2 axis has no effect on the proliferation ability of TNBC cells in breast cancer. However, in a co-culture system, miR-200c/PAI-2 increased IL-10 expression and secretion in TNBC cells, ultimately promoting M2 macrophage polarization (Meng et al., 2020). On the other hand, miR-720 can regulate the direction of macrophage polarization by regulating GATA3, a transcription factor involved in M2 macrophage polarization, and the downregulation of its expression inhibits M2 macrophage polarization, which further regulates TNBC progression (Zhong and Yi, 2016). Exosomal miR-934 from the colorectal cancer cell (CRC) downregulated PTEN expression and activated the PI3K/AKT signaling pathway to induce M2-type macrophage polarization, further affecting the ability of macrophages to mediate colorectal cancer progression (Zhao et al., 2020). Likewise, BCA-derived miR-222 activated the Akt signaling pathway and polarized M2 macrophages, promoting breast cancer cell progression in a feedback loop (Chen WX. et al., 2021). MiR-21 from bladder cancer directly downregulates PTEN and increases PI3K/Akt signaling activity while activating STAT3. The activation of STAT3 is the key to the differentiation of macrophages to the M2 phenotype, and the expression of M2-type signature genes promotes bladder cancer cell invasion and migration (Lin et al., 2020). In ovarian cancer, miR-217 inhibits M2 polarization by targeting IL-6 and modulating the JAK3/STAT3 signaling pathway (Jiang et al., 2019).

The lncRNA HOTAIR has been found to be dysregulated in many types of cancer, including colon, pancreas, liver, and gastrointestinal stromal tumors, and has been shown to contribute to their metastasis. Furthermore, HOTAIR promotes the polarization of macrophages towards the M2 phenotype, which is associated with tumor promotion and disease progression (Geng et al., 2011; Niinuma et al., 2012; Kim et al., 2013; Xue et al., 2015; Wang et al., 2022). In laryngeal squamous cell carcinoma, high expression of lncRNA HOTAIR can inhibit PTEN expression through PTEN methylation. si-lncRNA HOTAIR lentivirus transfection of LSCC cells can inhibit tumor cell proliferation, block cell growth, increase apoptotic cells, and significantly inhibit tumorigenesis in nude mice (Li et al., 2013).

In thyroid cancer, lncRNA MALAT1 inhibits the release of inflammatory cytokines TNF- and IL-12 by mediating FGF2 protein secretion by TAMs, promotes FTC133 cell proliferation, migration, and invasion, and induces angiogenesis (Huang et al., 2017). However, in HCC studies, MALAT1 has the opposite effect, and knockdown of MALAT1 inhibits VEGF-A production, impairs angiogenesis in HUVECs, and promotes the polarization of macrophages towards M1 (Hou et al., 2020). In gastric cancer cells, H19 inhibits glucose consumption primarily via the H19/miR-519d-3p/LDHA axis, promotes aerobic glycolysis, proliferation, and regulates immune cell activity, including T cells, Jurkat cells, and TAMs (Sun et al., 2021). The lncRNA RPPH1 has been found to induce EMT in CRC by interacting with β-III tubulin (TUBB3) to block its ubiquitination. RPPH1 can be transported to macrophages through exosomes, mediate the M2 polarization of macrophages, and promote the metastasis and proliferation of CRC cells (Liang et al., 2019). lncRNA LCC-HOXB8-1:2 is present in exosomes of neuroendocrine-differentiated colorectal cancer (CRC) cells and causes upregulation of CXCR3 expression in macrophages by binding to hsa-miR-6825-5p as a competitive endogenous RNA (ceRNA). Upregulated CXCR3 binds to the receptor CXCL10 on CRC, a process that leads to tumor-associated macrophage (TAM) infiltration and M2 polarization, thereby promoting the progression of neuroendocrine differentiated CRC (Li et al., 2022). In lung adenocarcinoma, LUAD cells released exosomes containing miR-19b-3p, which was transported to TAMs. miR-19b-3p then targets PTPRD, inhibits its-mediated STAT3 dephosphorylation and leads to STAT3 activation, thereby polarizing tam to the M2 subtype. Activated STAT3 induces transcription of LINC00273 in M2 macrophages. Exosomes containing LINC00273 were transferred to LUAD cells, where it recruited NEDD4 to promote ubiquitination and degradation of LATS2, and induced Hippo pathway inactivation to promote lung adenocarcinoma metastasis (Chen J. et al., 2021). Exosomal TUC339 from Hepatocellular carcinoma (HCC) regulates macrophage activation and polarization (Li et al., 2018). LINC00662 is another lncRNA that promotes M2 macrophage polarization and HCC progression by activating Wnt/-catenin signaling (Tian et al., 2020). Furthermore, overexpression of SNHG20 stimulates the activation of STAT6, induces M2 polarization of hepatic KCs, and promotes Non-alcoholic Fatty Liver Disease (NAFLD) progression to HCC (Wang et al., 2019). On the other hand, lncRNA FTX can inhibit the progression of NAFLD to HCC by regulating Kupffer cell M1/M2 polarization (Wu et al., 2020).

Numerous additional circRNAs have been discovered to affect immune responses by modifying macrophage polarization. CircRNA TCFL5 promotes esophageal cancer cell proliferation, invasion, and migration by regulating the FMNL2/miR-543 axis, mediates macrophage M2 polarization, and promotes tumor growth *in vivo* (Lin et al., 2022).

**FIGURE 4:**
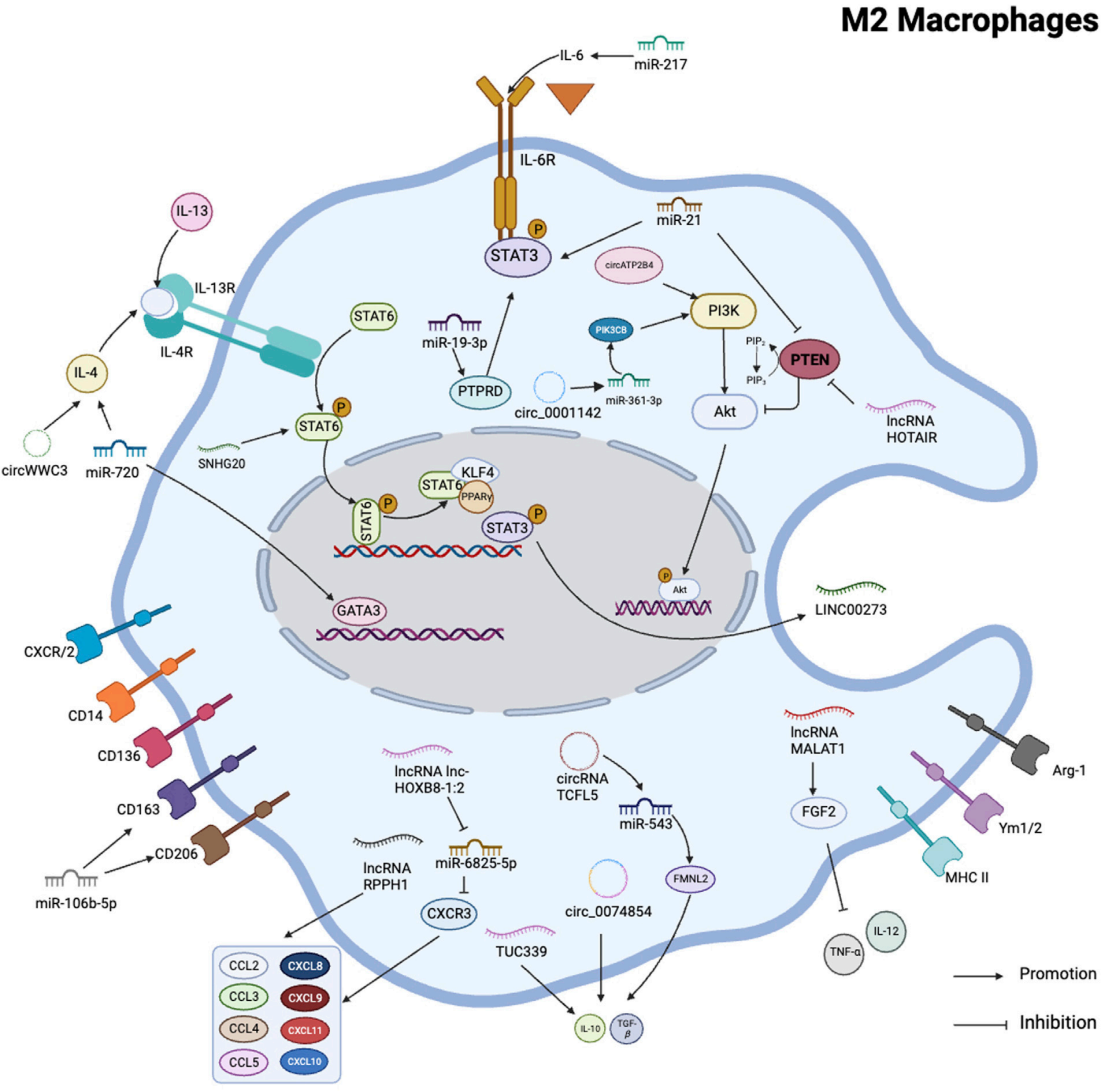
Schematic summary of the role of various non-coding RNAs in regulating M2 polarization in macrophages. This schematic illustrates the role of ncRNAs as key regulators of macrophage M2 activation and polarization in several human cancer cells. It iillustrates that PI3K/AKT pathway and STAT family were involved in macrophage M2 polarization.

CircATP2B4 regulates SREBF1 in ovarian cancer by binding to miR-532-3p, which not only reduces oxidative stress by increasing fatty acid synthesis but also promotes TAM polarization into M2. MiR-532-3p also promotes macrophage M2-type polarization via the PI3K/AKT signaling pathway (Liu et al., 2019; Wang et al., 2023). Exosomal circ 0001142 promoted the M2 polarization of macrophages and increased the proliferation and metastasis of breast cancer cells via the miR-361-3p/PIK3CB axis (Lu et al., 2022). CircWWC3 can increase IL-4 expression and secretion in breast cancer cells, and IL-4 binding to this receptor can activate PI3K and STAT6, as well as enhance M2-like polarization of macrophages in TME, and M2 further promotes breast cancer cells. The upregulation of IL-4 not only increased the expression of PD-L1 in breast cancer cells but also promoted the expression of PD-L1 in M2 macrophages, allowing for immune escape from breast cancer (Zheng et al., 2022). In hepatocellular carcinoma, hsa_circ_00074854 knockdown reduces HuR protein stability and inhibits ZEB1 signaling pathway to inhibit HCC cell migration, invasion, and EMT. Downregulation of hsa_circ_00074854 HCC exosomes inhibits HCC cell migration, invasion and EMT by inhibiting macrophage M2 polarization (Wang et al., 2021) (Figure 4).

## 5 Discussion and conclusion

The treatment of malignant tumors has always been a difficult problem. Conventional treatments mainly include surgery, chemotherapy, and radiotherapy. These drugs have poor stability and no targeting, and often have certain adverse reactions. While killing cancer cells, they also affect normal cells, with serious consequences as well. Macrophages are immune cells that aid in the identification and elimination of cancer cells. Noncoding RNAs can regulate macrophage activation and function in cancer. Directly or indirectly target key transcription factors and signaling pathways in macrophage polarization, or generate drug resistance to affect treatment. By modulating the expression and function of these RNA molecules, it is possible to reprogram macrophages from a pro-tumor state to an anti-tumor state, thereby improving cancer prognosis. The hottest research at present is to use exosomes with good biocompatibility and circulation stability as carriers to deliver drugs or inhibitors in the body to achieve anti-tumor effects. Exosomes are vesicles secreted by cells to the outside of the cell. They are 30–150 nm in size, have a double-layer membrane structure and saucer-like morphology, and contain rich inclusions (including nucleic acids, proteins and lipids, etc.), and participate in the molecular transfer between cells (Mondal et al., 2023).

However, exosomes themselves are not targeted, which limits the possibility of exosomes as carriers to carry drugs for targeted treatment of tumors.

Recently, researchers have invented a variety of targeted modification strategies for exosomes, so that drugs can not only be targeted for delivery, but also can prolong the circulation time of drugs in the blood, which makes it possible to treat cancer by targeting ncRNAs. The combination of exosomes and adenovirus can not only reduce the toxicity but also slow down the rapid clearance of AAV vectors. Adrienn et al. constructed the exosome-associated AAV vector exo-AAV9-CBA GFP to drive the expression of green fluorescent protein through specific promoters (NF-κB response promoter and truncated glial fibrillary acidic protein promoter), respectively. At the same time, peripheral gene expression is avoided, and these cells are subsequently used as the basis for the expression of secreted anti-tumor proteins to achieve therapeutic killing of tumor cells (Volak et al., 2018). Exosomes in combination with the CRISPR-Cas9 system can be targeted for gene editing. However, the intrinsic capability of natural exosomes is limited to the delivery of small molecules. Consequently, engineering exosomes becomes imperative to enhance the capacity and efficacy of macromolecule transportation. These tailored exosomes can be endowed with the capacity to selectively deliver their cargo to specific cellular or tissue targets. Ye et al. produced engineered exosomes (M-CRISPR-Cas9 exosomes) that efficiently loaded CRISPR-Cas9 components through protein-protein interactions, eliminating target genes more efficiently in A549 stop-DsRed reporter cells (Ye et al., 2020). The combination of exosomes and liposomes can not only reduce the toxicity of liposomes, but also encapsulate and deliver DNA macromolecules. Lv et al. innovatively developed a hybrid nanoparticle, denoted as genetically engineered exosome-thermosensitive liposome (gETL NP), which proficiently orchestrated the delivery of granulocyte-macrophage colony-stimulating factor (GM-CSF) and docetaxel, a chemical immunotherapy agent, to substantial tumor nodules. This strategic intervention markedly curtailed tumor progression. Furthermore, the integration of intraperitoneal hyperthermic chemotherapy (HIPEC) synergistically amplifies therapeutic efficacy (Lv et al., 2020).

In summary, the role of non-coding RNA in macrophage-mediated cancer is a rapidly developing research area, which may become a new target for treatment, and engineered exosomes may become a new way to treat cancer. However, more research is necessary to fully understand the complex mechanisms by which these RNA molecules regulate macrophage behavior and function in the context of cancer.
